# Stratified and combined analysis of the quality of lumbar spinal stenosis–related videos on major Chinese short video platforms

**DOI:** 10.3389/fdgth.2026.1769121

**Published:** 2026-05-04

**Authors:** Wenhui Zhao, Xinwei Chen, Zeda Wang, Zhehao Xiao, JiLin Yang, Zhipei Huang, Yanwei Jiang, Risheng Liang, Rui Wang

**Affiliations:** 1 Department of Neurosurgery, Fujian Medical University Union Hospital, Fuzhou, Fujian, China; 2 Fujian Institute of Neurosurgery, Fuzhou, Fujian, China; 3 Department of Ultrasonography, Fujian Medical University Union Hospital, Fuzhou, Fujian, China; 4 Fujian Medical University, Fuzhou, Fujian, China; 5 Department of Neurosurgery, Pingtan Comprehensive Experimental Zone Hospital, Pingtan, Fujian, China

**Keywords:** information quality, lumbar spinal stenosis, patient education, quality and reliability, short video

## Abstract

**Background:**

Lumbar spinal stenosis (LSS) is a degenerative disorder in which narrowing of the spinal canal compresses neural elements, causing pain, numbness, and limited mobility. With the rapid growth of Chinese short video platforms (TikTok, Bilibili, Xiaohongshu, Kwai, and WeChat), the public increasingly relies on short videos for LSS-related health information. However, the quality of such content has not been systematically evaluated.

**Methods:**

This cross-sectional content analysis searched each of five platforms using “lumbar spinal stenosis,” screened the top 100 results, and included 412 videos after applying predefined criteria. Basic characteristics and engagement metrics were extracted. Analyses were stratified by platform, uploader type, and video category. Video quality and reliability were assessed using the Global Quality Score (GQS), modified DISCERN (mDISCERN), and JAMA benchmark criteria. Spearman correlation analysis examined associations between video characteristics and quality scores.

**Results:**

High-quality content was concentrated on Bilibili and WeChat, particularly scientific explanations and professional course videos uploaded by healthcare professionals, whereas Kwai showed consistently low GQS scores across uploader types and categories. Video duration was moderately and positively correlated with GQS (*r* = 0.423), mDISCERN (*r* = 0.340), and JAMA scores (*r* = 0.357; all *p* < 0.001). Follower count and most engagement metrics (likes, saves, shares) showed only weak correlations with quality.

**Conclusions:**

Overall, the quality and reliability of LSS-related short videos on major Chinese platforms are suboptimal, with marked inter-platform variation. Content from non-professional uploaders and personal experience–focused videos tended to be of lower quality. Healthcare professionals and medical institutions should actively disseminate evidence-based LSS information via short video platforms, and viewers should preferentially seek credible, verifiable sources.

## 1 Introduction

LSS is a common degenerative spinal disorder and represents one of the leading causes of low back pain and neurogenic intermittent claudication in the aging population (1–5). Its prevalence increases markedly with age; studies have reported that radiographic LSS may affect up to 20%–30% of older adults, although symptomatic LSS accounts for a smaller but clinically significant proportion (6–8). In the context of modern lifestyles characterized by prolonged sitting, reduced physical activity, and poor posture, the potential burden of LSS appears to be rising (9–11). The chronic pain, mobility restriction, and functional impairment associated with LSS not only diminish individual quality of life but also increase healthcare utilization and impose substantial socioeconomic burden (12–14).

In the era of digital health, public pathways for acquiring medical knowledge have shifted from outpatient consultations and printed materials to the internet and social media (15). Previous research has shown that searching for health information online has become a common practice. Short video platforms such as TikTok, Bilibili, Xiaohongshu, Kwai, and WeChat are emerging as major entry points for information on diseases and medications, driven by algorithmic recommendations and high user engagement (16). Compared with traditional text-based materials or long-form popular science content, short videos present medical information through images, subtitles, voice-overs, and demonstrations. This multimodal format tends to attract greater attention, lower cognitive barriers, and facilitate the communication of complex medical concepts (17–19).

However, unlike academic journals, short video platforms lack formal peer review and strict regulatory mechanisms. As a result, the quality and reliability of health information on these platforms have been widely questioned (20–22). Studies focusing on liver cancer treatment (23), laryngeal cancer (24) and other conditions have reported that the overall scientific accuracy of related videos on TikTok and Bilibili is often suboptimal. Global Quality Score (GQS) and modified DISCERN ratings are frequently in the low-to-moderate range. Only a minority of videos produced by healthcare professionals can be considered relatively reliable, whereas many highly engaging videos rely mainly on personal experience and lack evidence-based support. These findings suggest that short video platforms have become important sources of health information, but they also carry structural risks related to uneven content quality and the rapid spread of inaccuracies.

Against this background, a systematic evaluation of the content coverage, quality, and reliability of LSS-related information on short video platforms is of clear importance. Such work can provide evidence to inform clinical health education, public decision-making, and platform regulation. To date, however, there has been no multi-platform analysis specifically focused on LSS. Consequently, it remains unclear how LSS-related videos on these platforms perform in terms of information quality and the extent to which they align with standards of evidence-based medicine. In this study, we analyzed LSS-related videos on major Chinese short video platforms, including TikTok, Bilibili, Xiaohongshu, Kwai, and WeChat. Using the GQS (25–27), mDISCERN (28, 29), and JAMA (30) benchmark criteria, we evaluated information quality and reliability across platforms, uploader types, and video categories (31). Our aim was to provide a comprehensive overview of the current landscape of LSS-related information and to generate empirical evidence to guide the optimization of health communication strategies and platform governance.

## 2 Methods

### 2.1 Ethical considerations

No ethics approval was applied for because the study only explored publicly available data on short video sharing platforms and did not conduct any experiments on human subjects. In addition, there is no identifiable information about individual users or IDs in this study.

### 2.2 Search strategy and data collection

According to the 2025 Analysis of China's Short Video Market Environment and User Behavior published by iiMedia Research (32), TikTok, Bilibili, Xiaohongshu, Kwai, and WeChat consistently ranked among the top five in terms of user usage and maintained high overall satisfaction scores. Consequently, these applications were identified as the five mainstream short-video platforms in China (33), and a systematic search was subsequently conducted across these sources to retrieve content pertaining to LSS. To reduce potential bias introduced by personalized recommendation algorithms, a new account was created and activated on each platform. On 13 October 2025, all platforms were searched within a single day using the standardized Chinese diagnostic term “腰椎管狭窄” (“lumbar spinal stenosis”). Abbreviations and colloquial expressions were deliberately excluded to ensure methodological consistency, minimize ambiguity, and enable standardized cross-platform comparisons, in line with previous studies evaluating online medical information quality.

For each platform, search results were reviewed in the platform's default ranking order, and the first 100 videos displayed at the time of the search were selected as candidate samples. The use of the “top 100 videos” has been validated and widely adopted in previous studies. These studies indicate that videos ranked beyond the top 100 do not materially affect overall analyses and that this range provides a reasonable representation of topic-related content on each platform.

**Figure 1:**
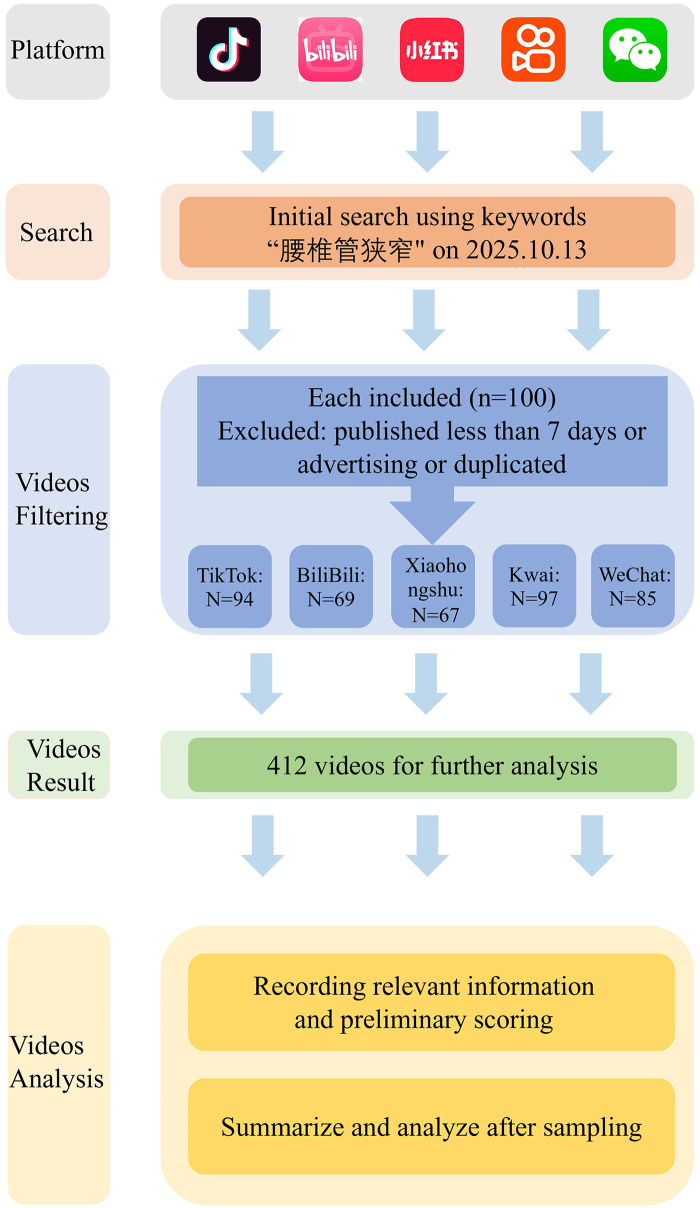
Search strategy and video screening procedure on lumbar spinal stenosis.

Videos were excluded if they met any of the following criteria: (1) not related to LSS; (2) duplicate content; (3) pure commercial advertising; (4) uploaded less than 7 days before the search date (Figure 1). After screening, the numbers of included videos were as follows: TikTok (*n* = 94); Bilibili (*n* = 69); Xiaohongshu (*n* = 67); Kwai (*n* = 97); WeChat (*n* = 85) yielding a total of 412 videos.

For each included video, we extracted basic information, including upload date, main topic, uploader type, video category, video duration, number of followers, and audience engagement indicators (numbers of likes, saves, comments, and shares). All data were entered into Microsoft Excel 2021 for subsequent descriptive and comparative analyses.

### 2.3 Classification of videos

Video classification and content evaluation were treated as two distinct analytical dimensions. Video category described the presentation style, whereas content domains referred to the medical topics addressed; a single video could include multiple content domains.

As shown in Table 1, uploaders were classified into four categories: (1) verified healthcare professionals; (2) lay users; (3) medical institutions; (4) organizations. The identities of healthcare professionals and medical institutions were verified using the official websites of their affiliated hospitals. The identities of lay users and organizations were determined based on platform verification information and relevant details shown in the videos.

**Table 1:** Detailed characteristics of videos.

Variables	Total (*n* = 412)	TikTok (*n* = 94)	BiliBili (*n* = 69)	Xiaohongshu (*n* = 67)	Kwai (*n* = 97)	WeChat (*n* = 85)	*P*
Likes, M (Q_1_, Q_3_)	970 (261, 3161)	2017 (1103,4316)	483 (64,3445)	669 (104,1978)	547 (173,1550)	1106 (332,11000)	<.001
Saves, M (Q_1_, Q_3_)	659 (166, 2726)	1776 (549,4660)	594 (145,5878)	460 (96,1925)	194 (48,723)	1,017 (222,9948)	<.001
Comments, M (Q_1_, Q_3_)	65 (18, 194)	80 (37,205)	66 (5,331)	41 (7,86)	76 (18,126)	76 (27,703)	0.003
Shares, M (Q_1_, Q_3_)	273 (67, 1488)	975 (305,2113)	135 (21,2526)	167 (74,725)	181 (52,639)	240 (60,2200)	<.001
Duration, M (Q_1_, Q_3_)	87 (50, 154)	81 (53,129)	363 (151,737)	71 (33,141)	76 (38,115)	78 (52,107)	<.001
Video releasetime, M (Q_1_, Q_3_)	84 (31, 214)	66 (39,130)	558 (107,974)	37 (15,87)	118 (46,229)	61 (30,183)	<.001
Followers (10^3^), M (Q_1_, Q_3_)	23 (4, 46)	35 (10,63)	23 (8,171)	16 (2,86)	22 (2,39)	NA (NA,NA)	<.001
GQS, M (Q_1_, Q_3_)	3 (2, 3)	3 (2, 3)	3 (3,3)	2 (2,3)	2 (1,2)	3 (2, 3)	<.001
mDISCERN, M (Q_1_, Q_3_)	3 (2, 3)	3 (3,3)	3 (3,3)	3 (2, 3)	2 (2,2)	3 (3,3)	<.001
JAMA score, M (Q_1_, Q_3_)	2 (2, 2)	2 (2,3)	2 (2,2)	2 (1,2)	2 (1,2)	2 (2,3)	<.001
Uploader type, *n* (%)							<.001*
Medical personnel	306 (74.27)	81 (86.17)	44 (63.77)	40 (59.70)	74 (76.29)	67 (78.82)	
Ordinary users	70 (16.99)	10 (10.64)	20 (28.99)	20 (29.85)	15 (15.46)	5 (5.88)	
Medical institutions	3 (0.73)	0 (0.00)	1 (1.45)	0 (0.00)	0 (0.00)	2 (2.35)	
Organizations	33 (8.01)	3 (3.19)	4 (5.80)	7 (10.45)	8 (8.25)	11 (12.94)	
Video category, *n* (%)							<.001*
Scientific Explanation	272 (66.02)	77 (81.91)	27 (39.13)	41 (61.19)	65 (67.01)	62 (72.94)	
Professional Courses or Lectures	9 (2.18)	0 (0.00)	8 (11.59)	0 (0.00)	1 (1.03)	0 (0.00)	
Personal or Collective Experiences	36 (8.74)	5 (5.32)	5 (7.25)	9 (13.43)	11 (11.34)	6 (7.06)	
Other Content	95 (23.06)	12 (12.77)	29 (42.03)	17 (25.37)	20 (20.62)	17 (20.00)	

GQS, global quality scale; Mdiscern, modified DISCERN; JAMA, Journal of the American Medical Association.

Video content was categorized into four types: (1) scientific explanations; (2) professional courses or lectures; (3) personal or group experiences; (4) other content.

### 2.4 Assessment of video content and quality

All videos were independently rated by two clinical experts (J.Y. and F.J.). Any disagreement between the two raters was resolved by a third reviewer (W.R.). All three assessors were front-line clinicians working in tertiary hospitals, each with more than 15 years of clinical experience. They used the Global Quality Scale (GQS), the modified DISCERN instrument (mDISCERN), and the Journal of the American Medical Association (JAMA) benchmark criteria to comprehensively evaluate the overall quality and reliability of each video. These tools are among the most commonly used instruments for assessing the quality of health information and have been widely applied on short video platforms.

The GQS was used to assess the accuracy, usability, coherence, and practical value of online information. It is a five-point scale commonly applied to evaluate the quality of patient education videos and has been widely adopted in previous studies. Scores range from 1 to 5, with higher scores indicating better video quality.

The mDISCERN is a modified version of the DISCERN tool and was used to assess the reliability and completeness of the information presented. DISCERN is a validated and reliable instrument for evaluating the quality of written consumer health information and helps patients and healthcare professionals distinguish higher-quality resources from lower-quality ones. Previous research has shown that the mDISCERN has good applicability and it has been widely used in many studies. The mDISCERN consists of five items; each “yes” response is scored as 1 and each “no” response as 0. Higher total scores indicate greater reliability of the video.

The JAMA benchmark criteria were used to evaluate the transparency and reliability of information disclosure. The four components are: (1) provision of authorship information; (2) clear listing of references or sources; (3) indication of the initial publication date and subsequent updates; and (4) disclosure of conflicts of interest, funding, sponsorship, advertising support, and ownership of the video. Each criterion fulfilled is awarded 1 point, yielding a total score ranging from 0 to 4.

In addition, video topics were classified into six domains: etiology, symptoms, diagnosis, prevention, treatment, and prognosis. Two raters evaluated the extent to which each video covered each domain using a three-point Likert scale: 0 (no related content), 1 (partial coverage), and 2 (comprehensive coverage) (Table 2). Detailed scoring criteria for GQS, mDISCERN, and JAMA are provided in the (Supplementary Tables S1–S2). The three experts discussed the scoring framework in advance and reached consensus on all definitions. During the rating process, they referred to and adhered to evidence-based clinical guidelines for LSS to ensure clinical validity and consistency of the assessments (3, 34–37).

**Table 2:** Subjective ratings of videos from different platforms.

Platforms Content		Tiktok	Bilibili	Xiaohongshu	Kwai	Wechat
Etiology, *N* (%)	No content	44 (46.81)	35 (50.72)	29 (43.28)	57 (58.76)	35 (41.18)
	Some content	33 (35.11)	17 (24.64)	27 (40.30)	40 (41.24)	38 (44.71)
	Extensive content	17 (18.09)	17 (24.64)	11 (16.42)	0 (0.00)	12 (14.12)
Symptoms, *N* (%)	No content	10 (10.64)	16 (23.19)	16 (23.88)	18 (18.56)	13 (15.29)
	Some content	49 (52.13)	21 (30.43)	21 (31.34)	67 (69.07)	17 (20.00)
	Extensive content	35 (37.23)	32 (46.38)	30 (44.78)	12 (12.37)	55 (64.71)
Diagnosis, *N* (%)	No content	5 (5.32)	15 (21.74)	12 (17.91)	23 (23.71)	3 (3.53)
	Some content	50 (53.19)	19 (27.54)	39 (58.21)	59 (60.82)	36 (42.35)
	Extensive content	39 (41.49)	35 (50.72)	16 (23.88)	15 (15.46)	46 (54.12)
Prevention, *N* (%)	No content	72 (76.60)	56 (81.16)	59 (88.06)	89 (91.75)	75 (88.24)
	Some content	15 (15.96)	8 (11.59)	7 (10.45)	8 (8.25)	8 (9.41)
	Extensive content	7 (7.45)	5 (7.25)	1 (1.49)	0 (0.00)	2 (2.35)
Treatment, *N* (%)	No content	3 (3.19)	26 (37.68)	20 (29.85)	17 (17.53)	15 (17.65)
	Some content	19 (20.21)	14 (20.29)	19 (28.36)	30 (30.93)	7 (8.24)
	Extensive content	72 (76.60)	29 (42.03)	28 (41.79)	50 (51.55)	63 (74.12)
Prognosis, *N* (%)	No content	22 (23.40)	17 (24.64)	23 (34.33)	42 (43.30)	32 (37.65)
	Some content	62 (65.96)	26 (37.68)	33 (49.25)	53 (54.64)	38 (44.71)
	Extensive content	10 (10.64)	26 (37.68)	11 (16.42)	2 (2.06)	15 (17.65)

### 2.5 Statistical analysis

The Shapiro–Wilk test was used to assess the normality of continuous variables. Non-normally distributed continuous variables were summarized as medians and interquartile ranges (IQRs). Categorical variables were presented as frequencies and percentages. Comparisons of non-normally distributed continuous variables between two groups were performed using the Mann–Whitney *U* test, and comparisons among three or more groups were conducted using the Kruskal–Wallis test. For categorical variables, the *χ*^2^ test, continuity-corrected *χ*^2^ test, or Fisher's exact test was applied as appropriate. Descriptive statistics were used to summarize content coverage stratified by platform and video type. Spearman rank correlation analysis was used to evaluate associations between video characteristics and video quality scores.

Inter-rater agreement between the two primary raters for GQS and mDISCERN scores was assessed using the kappa statistic, with a Cohen's *κ* ≥ 0.75 indicating good agreement.

All statistical analyses and visualizations were performed using R (version 4.5.1) and IBM SPSS Statistics (version 26.0). A two-sided *p* value < 0.05 was considered statistically significant. During the preparation of this manuscript, an artificial intelligence–based language model (ChatGPT, GPT-5.1 Thinking; OpenAI, San Francisco, CA, USA) was used only to assist with English language editing, including grammar checking and improving clarity and readability. All scientific content, study design, data analysis, and interpretation of results were conceived, written, and critically revised by the authors. The authors have carefully reviewed and verified all AI-assisted text and take full responsibility for the integrity and accuracy of the manuscript.

## 3 Results

### 3.1 Video characteristics

A total of 412 LSS-related short videos were included: TikTok (*n* = 94); Bilibili (*n* = 69); Xiaohongshu (*n* = 67); Kwai (*n* = 97); WeChat (*n* = 85). Overall, user engagement differed markedly across platforms (Table 1). TikTok and WeChat showed the highest levels of interaction. On TikTok, the median numbers of likes, saves, and shares were 2,017 [interquartile range (IQR): 1,103–4,316], 1,776 (IQR: 549–4,660), and 975 (IQR: 305–2,113), respectively. On WeChat, the median numbers of likes and saves were 1,106 (IQR: 332–11,000) and 1,017 (IQR: 222–9,948), both clearly exceeding those of the other platforms. In contrast, Xiaohongshu and Kwai showed comparatively low numbers of likes, saves, and shares.

The overall median video duration was 87 s (IQR: 50–154). Bilibili videos were substantially longer, with a median duration of 363 s (IQR: 151–737), whereas videos on the remaining platforms were typically around 1 min in length. The median number of followers per uploader was 23,000 (IQR: 4,000–46,000). TikTok and Bilibili accounts tended to have larger follower bases, whereas Xiaohongshu and Kwai uploaders had fewer followers.

With respect to uploader type, 74.3% of videos were posted by healthcare professionals and 17.0% by lay users; accounts belonging to medical institutions contributed only 0.7% of videos. Regarding video category, 66.0% were scientific explanation videos, 8.7% presented personal or group experiences, and only 2.2% were formal courses or lectures, which were concentrated mainly on Bilibili (Table 1).

Inter-rater agreement between the two primary raters was good to excellent for all scoring tools. Cohen's *κ* coefficients were 0.80 for the JAMA score, 0.81 for the mDISCERN score, and 0.84 for the GQS, indicating a high level of consistency (*κ* > 0.80).

### 3.2 Assessment of video content

Coverage of the six core domains is summarized in Table 2. For etiology, a large proportion of videos on Kwai (58.76%) and TikTok (46.81%) contained no relevant etiological information. In contrast, Bilibili performed best in the diagnostic domain, with 50.72% of videos providing more detailed diagnostic content. Symptom descriptions were most comprehensive on WeChat (64.71%) and Bilibili (46.38%).

**Figure 2:**
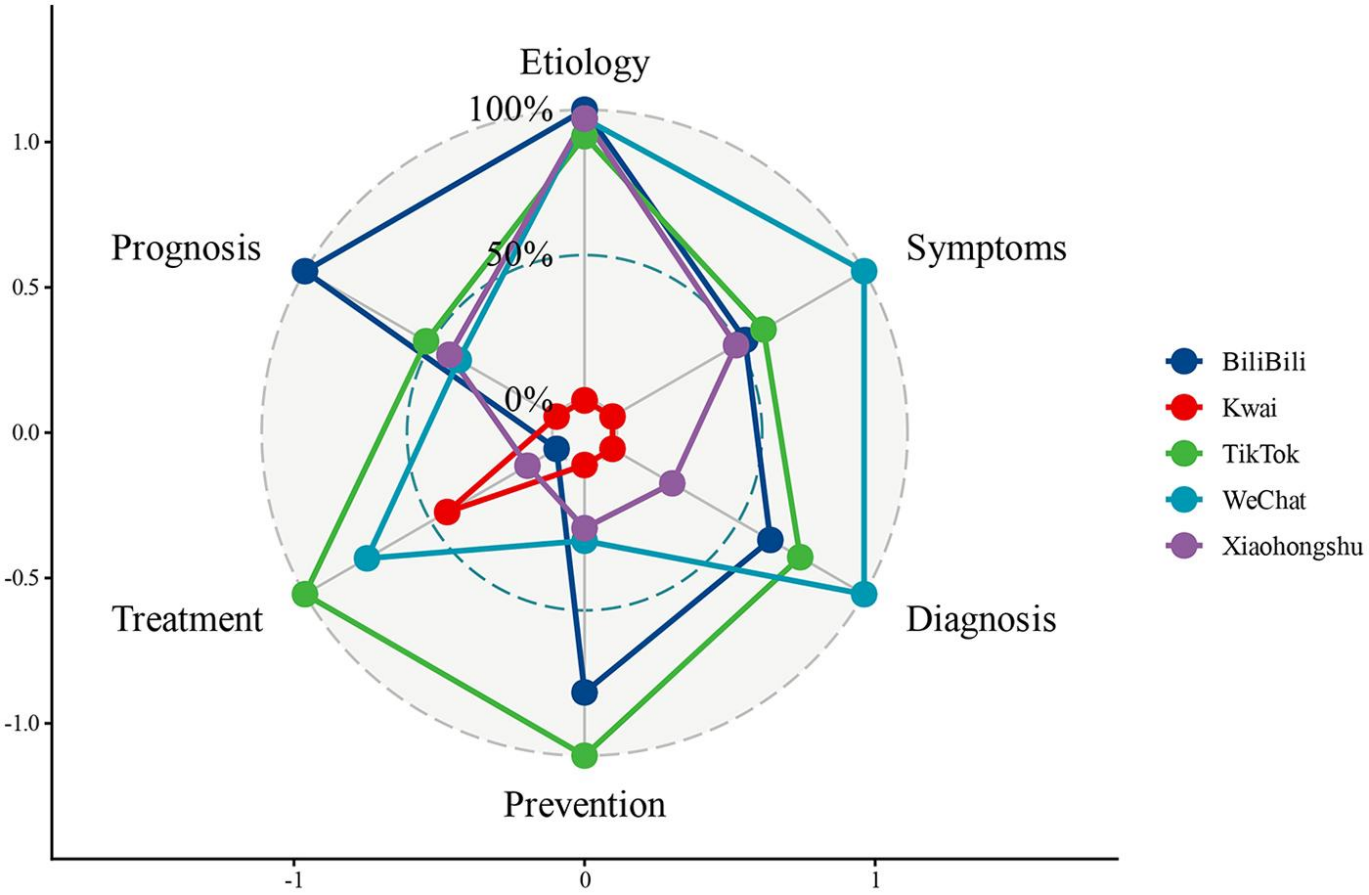
Radar map of content score.

Treatment was the most thoroughly covered topic across platforms. More than half of the videos on TikTok (76.60%), WeChat (74.12%), and Kwai (51.55%) provided detailed treatment-related information. By comparison, prevention was consistently underrepresented. On all platforms, fewer than 7.5% of videos achieved a “comprehensive” (score 2) rating for preventive content. The overall distribution of content coverage scores across all videos is shown in Figure 2.

### 3.3 Video quality and reliability assessment

#### 3.3.1 Quality and reliability by video platform

As shown in Table 1, videos on TikTok and Bilibili achieved the highest GQS and mDISCERN scores, with median values of 3 for both scales. In contrast, videos on Kwai had the lowest median scores across all three instruments (GQS: 2; mDISCERN: 2; JAMA: 2). WeChat videos demonstrated intermediate overall quality (GQS: 3) but relatively high reliability (mDISCERN: 3; JAMA: 2). Xiaohongshu videos were characterized by lower quality (GQS: 2) but acceptable reliability (mDISCERN: 3).

**Figure 3:**
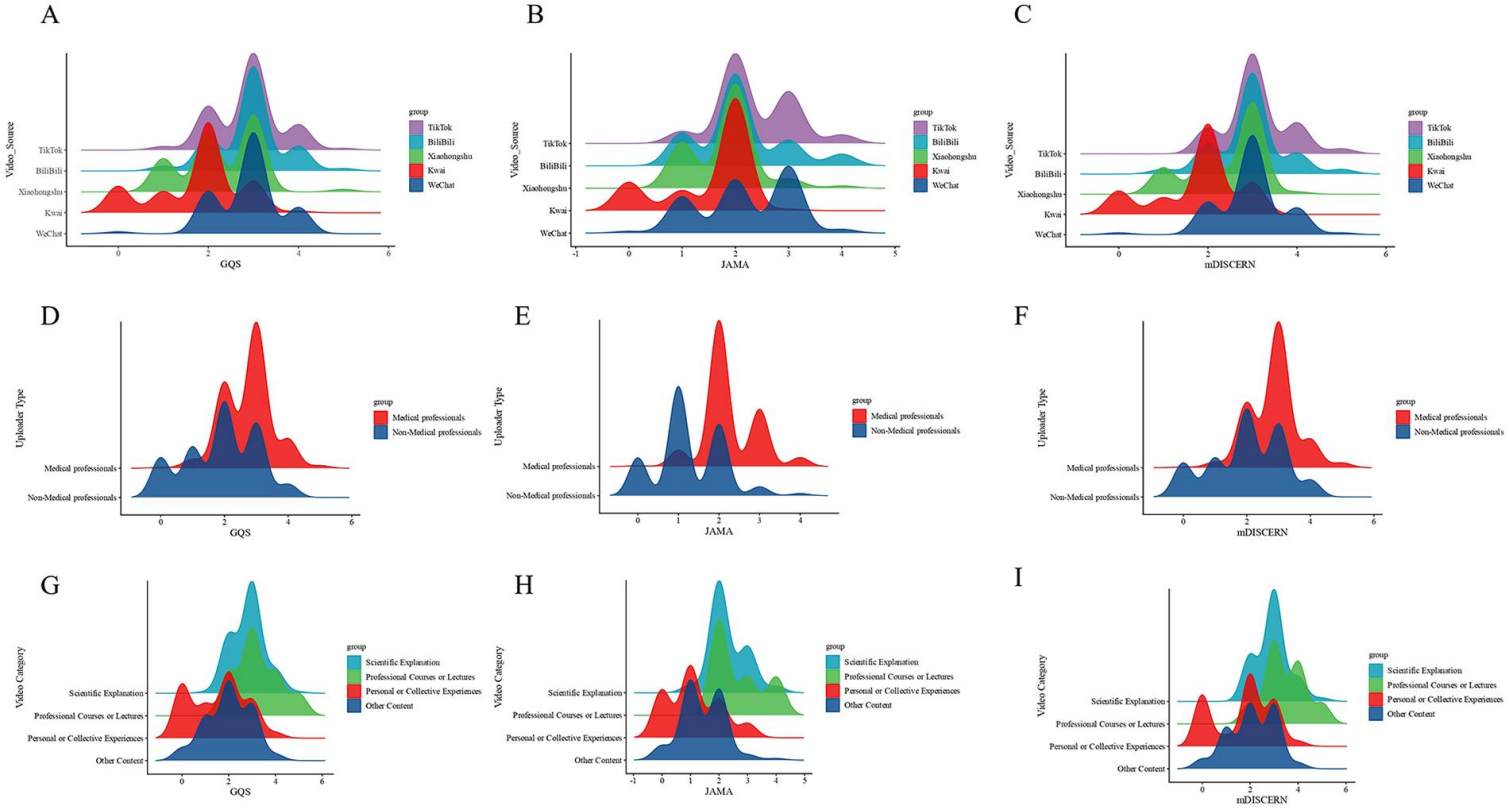
Comparison of the quality and reliability of lumbar spinal stenosis short videos on GQS, JAMA, mDISCERN. **(A–C)** different platforms; **(D–F)** different uploaders; **(G–I)** different video categories.

Ridgeline plots (Figures 3A–C) showed that the distributions of GQS and mDISCERN scores for TikTok and Bilibili were sharply peaked, right-shifted, and relatively concentrated, indicating consistently higher and more stable quality and reliability on these two platforms. In contrast, the distributions for Kwai were more dispersed and left-skewed, suggesting heterogeneous and generally lower-quality content.

#### 3.3.2 Quality and reliability by uploader type

To improve classification consistency, uploaders were grouped into healthcare professionals (including individual clinicians and medical institutions) and non-professionals (including lay users and non-medical organizations) for analysis (Table 3). Videos uploaded by healthcare professionals had significantly higher scores on all three scales [GQS: 3 (2–3); mDISCERN: 3 (2–3); JAMA: 2 (2–3)] compared with those uploaded by non-professionals [GQS: 2 (1–3); mDISCERN: 2 (1–3); JAMA: 1 (1–2)].

**Table 3:** Different uploader video ratings.

Uploader types	Medical personnel	Ordinary users	Medical institutions	Organizations	*P*
GQS, M (Q_1_, Q_3_)	3.00 (2.00,3.00)	2.00 (1.00,3.00)	3.00 (3.00,3.50)	2.00 (2.00,3.00)	<.001
mDISCERN, M (Q_1_, Q_3_)	3.00 (2.00,3.00)	2.00 (1.00,3.00)	3.00 (3.00,3.50)	2.00 (2.00,3.00)	<.001
JAMA score, M (Q_1_, Q_3_)	2.00 (2.00,3.00)	1.00 (1.00,2.00)	3.00 (3.00,3.00)	1.00 (1.00,2.00)	<.001

Ridgeline plots (Figures 3D–F) further demonstrated that score distributions for healthcare professionals were clearly right-shifted and more concentrated for all three metrics, indicating higher and more consistent quality and reliability. In contrast, distributions for non-professionals were left-shifted and more dispersed, reflecting generally lower and more variable content quality. Together, these findings underscore uploader professional background as a key determinant of informational reliability.

#### 3.3.3 Quality and reliability by video category

Analyses stratified by video category (Table 4) showed that scientific explanation videos [GQS: 3 (2–3); mDISCERN: 3 (2.75–3); JAMA: 2 (2–3)] and professional courses or lectures were associated with the highest quality and reliability scores. In contrast, videos based on personal or group experiences received the lowest ratings [GQS: 2 (0–2); mDISCERN: 2 (0–3); JAMA: 1 (0–2)].

**Table 4:** Rating for different video categories.

Video category	Scientific explanation	Professional courses or lectures	Personal or collective experiences	Other content	*P*
GQS, M (Q_1_, Q_3_)	3.00 (2.00,3.00)	3.00 (3.00,4.00)	2.00 (0.00,2.00)	2.00 (1.00,3.00)	<.001
mDISCERN, M (Q_1_, Q_3_)	3.00 (2.75,3.00)	3.00 (3.00,4.00)	2.00 (0.00,3.00)	2.00 (1.00,3.00)	<.001
JAMA score, M (Q_1_, Q_3_)	2.00 (2.00,3.00)	2.00 (2.00,3.00)	1.00 (0.00,2.00)	1.00 (1.00,2.00)	<.001

Ridgeline plots (Figures 3G–I) revealed that scientific explanation videos exhibited the most prominent, right-shifted peaks across all three indicators, indicating the best and most stable performance. By comparison, personal experience videos showed the most dispersed and left-skewed distributions, again pointing to lower and less consistent quality.

#### 3.3.4 Combined effects of platform, uploader type, and video category

**Figure 4:**
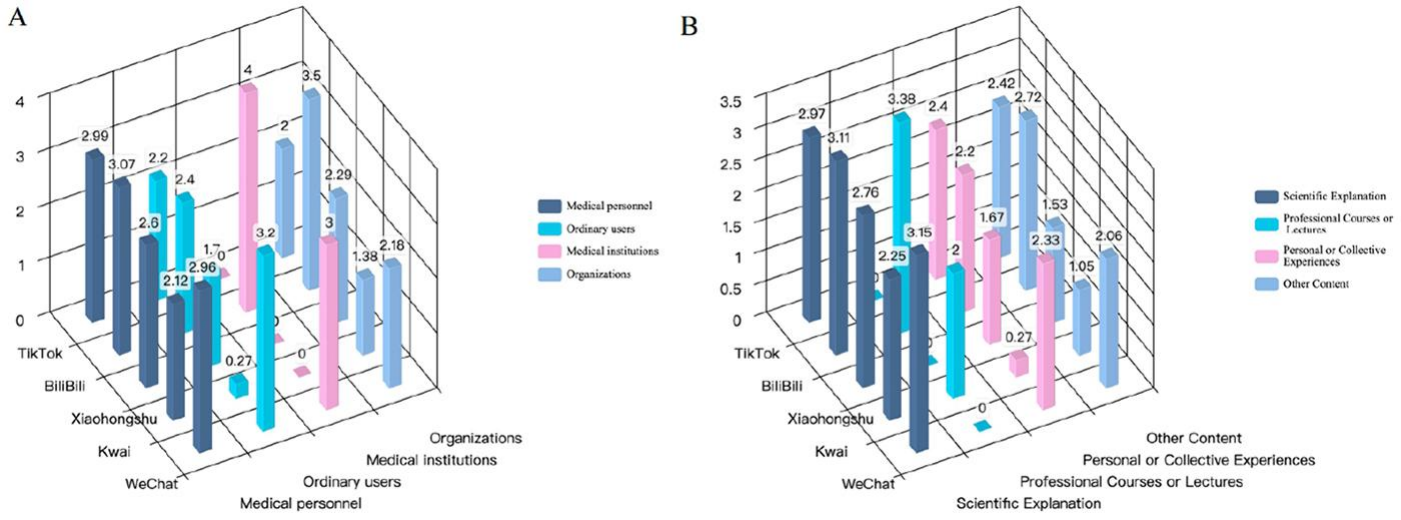
**(A)** The combined effect of platform and uploader type on mean GQS scores; **(B)** the combined effect of platform and video category.

Three-dimensional bar charts were used to further examine the combined effects of platform, uploader type, and video category on video quality. Figure 4A illustrates the joint effect of platform and uploader type on mean GQS scores. Among healthcare professional uploaders, Bilibili showed the best performance (individual clinicians: 3.07; medical institutions: 4.00), while WeChat maintained a consistently high level of quality (healthcare professionals: 2.96). On Kwai, only videos from healthcare professionals reached modest quality (2.12), whereas those from lay users received very low mean scores (0.27).

Figure 4B presents the combined effect of platform and video category. Bilibili showed a clear advantage for knowledge-intensive content (scientific explanations: 3.11; professional courses: 3.38). WeChat also performed well in scientific explanation videos (3.15). In contrast, Kwai performed poorly across all content categories, with personal experience videos again scoring particularly low (0.27). These patterns indicate that video quality is jointly shaped by platform characteristics, uploader professional background, and content type.

### 3.4 Correlation analysis

Spearman correlation analyses (Table 5) showed that video duration was moderately and positively correlated with all three quality and reliability scores (GQS: *r* = 0.423; mDISCERN: *r* = 0.340; JAMA: *r* = 0.357; all *p* < 0.001). This suggests that longer videos are more likely to provide more comprehensive and systematic medical information.

**Table 5:** Spearman correlation.

Quality score	Duration		Likes		Video releasetime		Comments		Saves		Shares		Followers	
	*r*	*p* value	*r*	*p* value	*r*	*p* value	*r*	*p* value	r	*p* value	*r*	*p* value	*r*	*p* value
GQS score	0.423	0	0.074	0.135	−0.057	0.252	−0.037	0.452	0.151	0.002	0.125	0.011	0.128	0.021
mDiscern score	0.34	0	0.061	0.216	−0.09	0.069	−0.048	0.328	0.133	0.007	0.095	0.054	0.032	0.0561
JAMA score	0.357	0	0.099	0.044	−0.157	0.001	−0.024	0.628	0.119	0.015	0.115	0.02	0.243	0

GQS, global quality scale; JAMA, Journal of American Medical Association.

The number of followers was weakly but significantly correlated with the JAMA score (*r* = 0.243, *p* < 0.001). Its correlation with the GQS was statistically significant but of very small magnitude (*r* = 0.128). By contrast, traditional engagement metrics (likes, comments, saves, and shares) showed only weak correlations with quality and reliability scores. Even when statistically significant, most correlation coefficients remained in the small-effect range, and there was virtually no meaningful association between the number of comments and any quality metric.

Overall, these findings indicate that video “popularity” is only loosely linked to scientific quality and reliability. Engagement volume is therefore a poor surrogate for the quality of health information in short videos.

## 4 Discussion

LSS is a degenerative narrowing of the spinal canal that compresses the nerve roots or cauda equina and presents with low back and leg pain, numbness, and reduced mobility (2, 38). It can lead to buttock pain with radiation to the lower limb and, in severe cases, substantially impair daily functioning and quality of life (39–41). At the same time, short video social media platforms such as TikTok, Bilibili, Xiaohongshu, Kwai, and WeChat have rapidly become important sources of health information for the public, owing to their concise and fragmented presentation, wide reach, and high level of user engagement (15). However, the convenience of this mode of dissemination is accompanied by uneven information quality, limited professional verification, and the rapid spread of potentially misleading content (15, 32, 42, 43). A systematic evaluation of the quality of LSS-related information on short video platforms is therefore of practical importance for clinical health education, public awareness, and platform regulation (20, 44–46). To our knowledge, this study provides the first comprehensive multi-platform evaluation of the quality and content coverage of LSS-related short videos across major Chinese short-video platforms.

In this study, we analyzed 412 LSS-related short videos and assessed video characteristics, content coverage, quality, and reliability from multiple perspectives. Substantial differences in video duration and engagement metrics were observed across platforms. TikTok recorded the highest numbers of likes, saves, and shares, with WeChat also showing relatively high engagement, whereas videos on Bilibili were notably longer in duration. Most of the included videos were uploaded by healthcare professionals (74.27%), and scientific explanation was the dominant content type (66.02%). Nevertheless, coverage of prevention-related topics was consistently poor across all platforms, with comprehensive prevention content observed in fewer than 7.5% of videos. In terms of overall quality, videos on TikTok and Bilibili achieved relatively higher GQS, mDISCERN, and JAMA scores, whereas those on Kwai scored lowest; WeChat and Xiaohongshu fell in the intermediate range. These findings highlight platform-level differences in engagement patterns, content structure, and baseline quality, and provide a foundation for more detailed stratified analyses by uploader type and video category. Even in a context where professional uploaders are prevalent and scientific explanation content is dominant, LSS-related short videos still show clear information gaps in prevention, long-term management, and prognosis. This suggests that the involvement of healthcare professionals does not automatically guarantee comprehensive or well-balanced content, and that the allocation of limited video time across different thematic domains remains a weak point in current health short video production.

Further stratified analyses showed that video quality and reliability were closely linked to uploader type and video category. Videos produced by healthcare professionals outperformed those from non-professional uploaders in accuracy, evidentiary support, and transparency of information sources. Scientific explanation and professional course or lecture videos also consistently received higher scores than personal or group experience–based content. In combination with the cross-tabulations presented in Supplementary Tables S1, S2 and the three-dimensional distributions in Figure 4, a clear structural pattern emerges: on Bilibili and WeChat, scientific explanation and professional course videos uploaded by clinicians and medical institutions account for a relatively large proportion of content and achieve more concentrated, higher quality scores; in contrast, on Kwai and Xiaohongshu, videos uploaded by lay users and social organizations, particularly those focused on personal experiences or “other” content, represent a larger share and generally receive lower quality and reliability ratings. These results indicate that low-quality content is not evenly distributed across platforms but instead shows platform-specific clustering. This pattern is consistent with previous work on the uneven quality of health information on social media and underscores the need to strengthen quality control mechanisms and trustworthy-source labeling in the short video environment.

Correlation analyses demonstrated moderate positive associations between video duration and all three quality and reliability metrics (GQS: *r* = 0.423, *p* < 0.001; mDISCERN: *r* = 0.340, *p* < 0.001; JAMA: *r* = 0.357, *p* < 0.001), suggesting that longer videos are more likely to provide comprehensive medical information. This is in line with earlier findings that adequate time is necessary to explain complex medical topics in depth. By contrast, the number of followers showed only a weak but statistically significant correlation with the JAMA score (*r* = 0.243, *p* < 0.001), and traditional engagement indicators (likes, comments, shares) displayed minimal correlations with quality metrics. The divergence between popularity and quality implies that high engagement cannot be assumed to reflect high-quality content. From a platform governance perspective, relying solely on “heat” or popularity to surface health-related content may unintentionally amplify low-quality information. It may therefore be necessary to incorporate quality-sensitive weighting or trusted-source labels into recommendation algorithms.

This study has several strengths. First, it covers five major Chinese short video platforms with a relatively large sample size (*n* = 412), providing a more comprehensive picture of the current digital communication environment for LSS-related information than studies limited to a single platform or small samples. Second, we combined three complementary assessment tools: GQS, mDISCERN, and JAMA, and conducted stratified and joint analyses by platform, uploader type, and video category. This design allowed us to identify multiple factors that shape video quality in a more systematic way. Based on these findings, the results are practically relevant for platform operators, healthcare and public health professionals, and policymakers. Platforms may consider increasing the algorithmic weight of scientific explanation and professional course content produced by healthcare professionals, while medical institutions and academic bodies can strategically address current gaps in topics such as prevention and long-term management of LSS to jointly improve the overall health information ecosystem.

Several limitations should be acknowledged. First, videos were sampled within a single time window using a fixed search strategy based on the standardized diagnostic term “lumbar spinal stenosis”, which may not fully capture the dynamic evolution of content under continuously changing platform algorithms or videos using non-standard, colloquial terminology. Second, although GQS, mDISCERN, and JAMA are widely used instruments and inter-rater agreement was high, quality assessment relied on subjective rating scales. Third, only Chinese-language platforms and content were included; therefore, the findings primarily reflect the Chinese context and may not be generalizable to other languages or regions. Finally, given the rapid updating of short-video content and engagement metrics, a single cross-sectional assessment provides only a snapshot. Future studies should integrate natural language processing and machine learning approaches to enable continuous, automated, and large-scale monitoring of health information quality on short-video platforms.

## 5 Conclusion

This study systematically evaluated the quality and reliability of lumbar spinal stenosis–related short videos on major Chinese short video platforms. Overall, video quality ranged from low to moderate, with marked differences between platforms. TikTok and Bilibili achieved relatively higher scores, while prevention-related content was consistently underrepresented across all platforms. Stratified and combined analyses further showed that high-quality content was mainly concentrated on Bilibili and WeChat, particularly in scientific explanation and professional course videos uploaded by healthcare professionals. In contrast, GQS scores on Kwai were low across uploader types and video categories, indicating that the interplay between platform characteristics and uploader type strongly shapes the quality of information ultimately reaching viewers.

In light of these findings, healthcare professionals and medical institutions should be encouraged to actively use short video platforms to provide high-quality, evidence-based educational content on lumbar spinal stenosis, with particular emphasis on prevention and long-term management. Platform operators should strengthen content review and trusted-source labeling, increase the recommendation weight of high-quality health information, and restrict the spread of clearly misleading content. Viewers should pay close attention to information sources and the professional background of uploaders, and should rely on qualified clinicians when making diagnostic and treatment decisions, in order to reduce health risks arising from low-quality or inaccurate information.

## Data Availability

The raw data supporting the conclusions of this article will be made available by the authors, without undue reservation.
