# Supplementary material for: Stratified and combined analysis of the quality of lumbar spinal stenosis–related videos on major Chinese short video platforms
Source: Front Digit Health. 2026 May 4;8:1769121. doi: 10.3389/fdgth.2026.1769121 (PMC13180894; doi:10.3389/fdgth.2026.1769121)
Supplement: Supplementary file 1 [file Table1.docx]

|  | TikTok | BiliBili | Xiaohongshu | Kwai | WeChat |
| --- | --- | --- | --- | --- | --- |
| Medical personnel | 2.99 | 3.07 | 2.60 | 2.12 | 2.96 |
| Ordinary users | 2.20 | 2.40 | 1.70 | 0.27 | 3.20 |
| Medical institutions | 0.00 | 4.00 | 0.00 | 0.00 | 3.00 |
| Organizations | 2.00 | 3.50 | 2.29 | 1.38 | 2.18 |

**Supplement table 1** The quality differences among different uploaders on various platforms
