# Supplementary material for: Stratified and combined analysis of the quality of lumbar spinal stenosis–related videos on major Chinese short video platforms
Source: Front Digit Health. 2026 May 4;8:1769121. doi: 10.3389/fdgth.2026.1769121 (PMC13180894; doi:10.3389/fdgth.2026.1769121)
Supplement: Supplementary file 2 [file Table2.docx]

|  | TikTok | BiliBili | Xiaohongshu | Kwai | WeChat |  |
| --- | --- | --- | --- | --- | --- | --- |
| Scientific Explanation | 2.97 | 3.11 | 2.76 | 2.25 | 3.15 |  |
| Professional Courses or Lectures | 0.00 | 3.38 | 0.00 | 2.00 | 0.00 |  |
| Personal or Collective Experiences | 2.40 | 2.20 | 1.67 | 0.27 | 2.33 |  |
| Other Content | 2.42 | 2.72 | 1.53 | 1.05 | 2.06 |  |

**Supplement table 2** The quality differences among different video categories on various platforms
